# An Investigation of a Novel Dural Repair Device for Intraoperative Incidental Durotomy Repair

**DOI:** 10.3389/fsurg.2021.642972

**Published:** 2021-06-30

**Authors:** Kartik Shenoy, Chester J. Donnally, Evan D. Sheha, Krishn Khanna, Srinivas K. Prasad

**Affiliations:** ^1^Rothman Institute at Thomas Jefferson University, Philadelphia, PA, United States; ^2^Institute for Spine Care, Chicago Institute of Neurosurgery and Neuroresearch, Rush Presbyterian Medical Center, Chicago, IL, United States; ^3^Departments of Orthopaedic Surgery and Neurological Surgery, Thomas Jefferson University and The Rothman Institute, Philadelphia, PA, United States

**Keywords:** incidental durotomy, dural tear, lumbar spine, surgical education, spine simulation

## Abstract

Incidental durotomies, or dural tears, can be very difficult and time consuming to repair properly when they are encountered in confined spaces. A novel dural repair device was developed to address these situations. In this paper, the novel device was assessed against the use of traditional tools and techniques for dural repairs in two independent studies using an intricate clinical simulation model. The aim was to examine the results of the two assessments and link the outcomes to the clinical use of the novel device in the operating room. The novel device outperformed conventional techniques as measured by dural repair time, CSF leak pressure and nerve root avoidance in the simulation. The results were generally replicable clinically, however, numerous additional clinical scenarios were also encountered that the simulation model was unable to capture due to various inherent limitations. The simulation model design, potential contributors to watertightness, clinical experiences, and limitation are discussed.

## Introduction

Dural tears are one of the most common complications in spine surgery (1, 2). The risk of an incidental durotomy increases with numerous factors including patient age and surgical complexity (3). Dural tears resulting in a persistent cerebrospinal fluid (CSF) leak may result in subsequent operations and substantial decreases in patient quality of life, in addition to imposing an economic burden on the healthcare system. A primary mechanical repair of the dura using a suture is the most effective way to prevent persistent CSF leaks (4, 5), however, achieving such a repair can be difficult when access and visualization are limited, such as in minimally invasive exposures, or when tears occur in less accessible locations in open procedures. In these situations, the use of traditional suturing techniques can be technically difficult, time consuming, and may necessitate additional decompression, leading to potential destabilization of the spine, and rendering the repair practically impossible. The inherent limitations of traditional suturing instruments prompted the creation of a novel dural repair device.

The novel device, DuraStat (DuraStat LLC, Exton PA, USA), deploys a double armed suture in a controlled manner through the dura to facilitate repairs in difficult clinical scenarios. Our investigation examined the potential benefits of this novel device as compared to conventional (conv.) instrumentation broadly used today. The conventional and novel repair techniques were compared over the course of two independent bench-top evaluations to assess time to gain initial control of the dural tear, overall time, safety and quality of the closure. The authors then used the novel device intraoperatively to examine if the findings of the study were applicable in the operating room.

## Materials and Methods

The evaluation of the novel approach compared to conventional techniques was conducted in three separate parts: Parts A and B were completed with a highly controlled simulated surgical environment and Part C evaluated the novel device in the clinical environment (Table 1).

**Table 1:** Three stages of investigational research for the novel dural repair device.

**Part A: Clinical simulation**	**Part B: Clinical simulation**	**Part C: Operating room**
Ten participants comparing novel dural repair device & traditional suture techniques	Ten participants comparing novel dural repair device & traditional suture techniques	The authors evaluated their clinical experiences using the novel device intraoperatively against the benchtop results
Primary endpoints: time to gain control of the tear by bringing the center of defect edges together using the initial suture	Primary endpoints: time to complete the total repair with two sutures, quality of repair and neurological safety (entrapped nerve roots)	Qualitative assessment of the realization of the experience captured in the simulated environment

In Part A, surgeons were timed to pass and secure the first suture of a repair to gain control of the dural tear by approximating the tissue at the center of the defect. In Part B, surgeons were timed to complete a two-stitch repair. Dural sac pressure readings were collected pre- and post-repair, and the dural sac from each repair was dissected to examine the degree of nerve root entrapment and to capture other notable repair attributes.

Part C of the study was conducted at two university hospitals. Part C evaluated the surgeon's experience using the novel device in numerous open, mini-open and minimally invasive procedures where incidental durotomies were encountered to assess if the benchtop experience with the novel approach would translate to the operating room environment.

**Figure 1:**
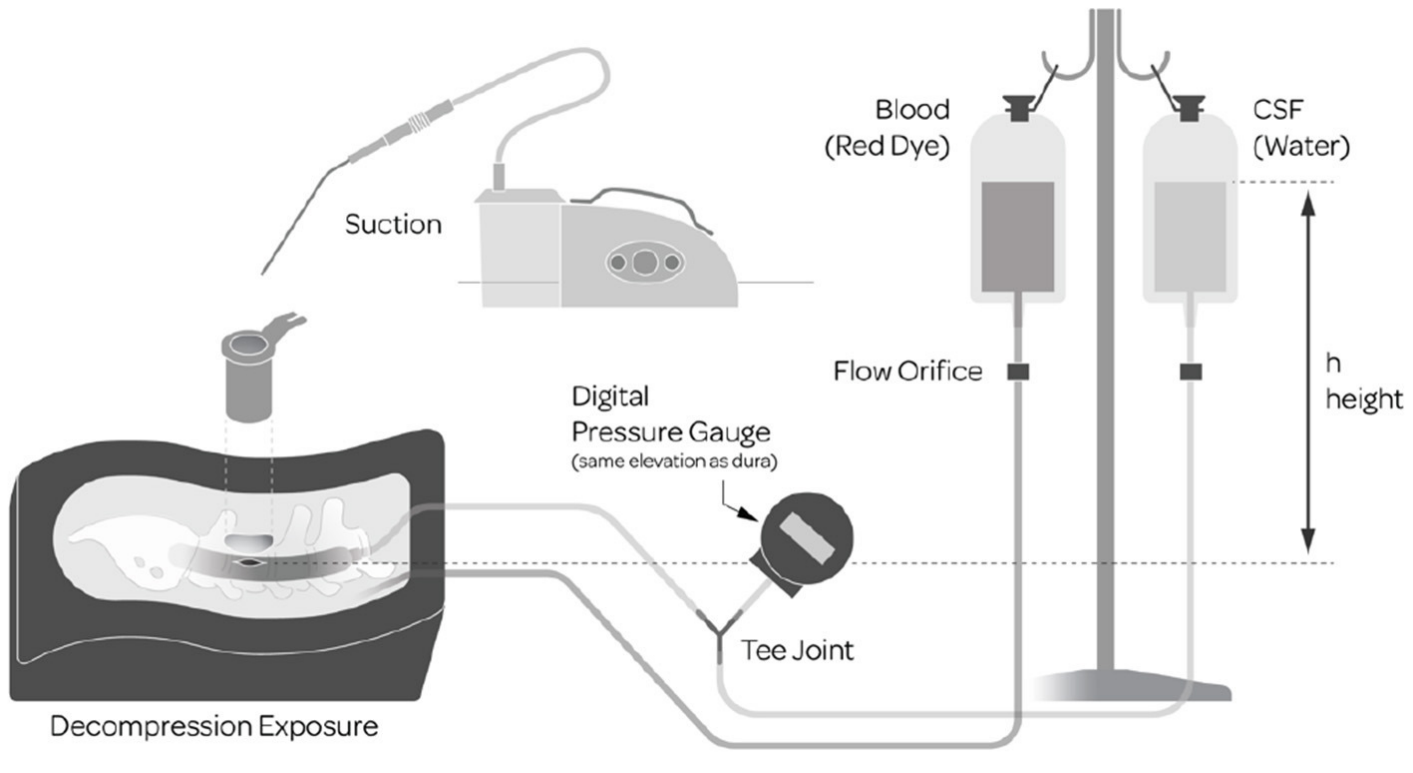
Simulated durotomy model.

The model used for the bench-top portions of the study, Parts A and B, was built upon a previously validated Sawbones model (6) SKU: 1324-62 (Pacific Research Company, Vashon Island, WA, USA) consisting of realistic synthetic anatomy (Figure 1). Numerous enhancements were added to the model for the purposes of this study to more closely emulate the intraoperative environment that surgeons face. The enhancements included the addition of simulated CSF and blood which required surgeons to suction away excess liquid to maintain visualization. The dural sac contained simulated nerve roots made from bundles of synthetic and elastic thread which could herniate out of the dural sac to add the dimension of nerve root management. Access to the repair site was through a 6 cm long, 22 mm diameter tubular retractor, at a depth of 4 cm and a standardized midline dural defect (7 mm linear tear) was made in the center of the exposure. To achieve realistic flowing CSF and blood, two separate IV bags were filled with 1,000 ml of water, one with the addition of red dye to simulate blood. Both bags were raised so that the top water level was at 30 cm above the dural element. A digital pressure gauge measured the hydrostatic characteristics of the dural sac. The water from the CSF bag flowed from inside the dural sac and the simulated blood flowed into the synthetic epidural space adjacent to the durotomy in the simulated surgical field. For the second comparative evaluation, the water bag was refilled and rehung to yield a direct before and after comparison to assess the quality of repair.

For Parts A and B, 10 surgeons completed two independent simulated surgical evaluations, with some population overlap between the surgeon groups (fifteen unique participants). Surgeons consisted of orthopedic and neurosurgery residents, fellows and attendings from one of the university hospitals. Participants were selected to represent numerous levels of training and experience.

Surgeons conducted one repair with conventional instrumentation and another with the novel dural repair device under loupe magnification. For the conventional repair, surgeons elected to use either a 6” locking Castroviejo (Integra LifeSciences Princeton, NJ, USA) or a 6” micro-pituitary (Boss Instruments, Gordonsville, VA, USA) to drive the needle and suture. The suture was a 6-0 Gore-Tex suture (Gore Medical Inc., Flagstaff, AZ, USA). A Medtronic 90° short straight ball tipped probe (Medtronic, Minneapolis, MN, USA) was also supplied to facilitate tissue identification and knot pushing.

### Statistical Analysis

Descriptive statistics of observations (N), means/medians, standard deviations (SD), minimum (MIN) and maximum (MAX) values for continuous variables and sample sizes (N) were used to compare the novel repair device and conventional methods. The null hypothesis was that there is no difference between the novel repair device and conventional methods. Analysis of Variance were used to compare the two methods for the time to place and tie the initial suture. A lower time reflects a superior method. Parametric paired-samples *t*-test were used to compare overall repair time, again a lower time reflects a superior method. Parametric paired-samples *t*-test were used to compare Quality of Repair between the two methods. Quality of repair was determined by the change in pressure in cm of H_2_O as read by a digital pressure gauge. Pressure before the repair minus pressure after the repair, where a smaller differential in pressure indicates a higher quality repair. Parametric paired-samples *t*-test were used to compare accuracy of suture placement as defined by gaps present after the repair and if repaired edges were everted. Patient safety as assessed by nerve root entrapment was calculated using McNemar's test. This method is most appropriate because it compares entrapment in one or both of the methods vs. totals across both methods. All statistical tests were two-sided at significance level alpha = 0.05. *P*-values were reported to three decimal places (0.000).

## Results

### Part A

For the first benchtop model, comparison focused on the time to gain initial control of the tear by bringing the center of edges of the tear together with the initial suture. The novel device took 57% less time to place and tie the initial suture as compared to the conventional technique, with less variation in times overall. The result was statistically significant (Novel: 3.38 min. ± 1.00, Conv.: 7.91 min. ± 4.48; *p* = 0.013) (Table 2). These results indicate that the novel device was reproducible in saving time, regardless of the participant's level of training, even immediately after being introduced to the technology for the first time.

**Table 2:** Repair time.

	**Novel dural repair device**	**Conventional suture technique**	***P*-value**
Part A: Time to first stitch	3.38 min. ± 1.00	7.91 min. ± 4.48	*p =* 0.013
Part B: Total repair time	7.03 min. ± 2.20	11.01 min. ± 4.44	*p =* 0.019

A survey of surgeon experience from Part A was also completed by each participant. 89% of participants noted that the novel device was perceived to be safer than conventional techniques, and 100% confirmed the novel device was easy to use. One participant did not follow the study protocol and their results were removed.

### Part B

For the second benchtop comparison focusing on total repair time, quality, and safety of the repair, the overall time to repair was again faster with the novel device than the conventional technique, taking 36% less time to complete the full repair. The results were statistically significant (Novel: 7.03 min. ± 2.20, Conv.: 11.01 min. ± 4.44; *p* = 0.019). The quality of repair was also significantly higher with the novel device as measured by the change in fluid pressure back to baseline (30 cm H_2_O) after the repair (Novel: 22.9 cm H_2_O ± 5.26, Conv.: 13.3 cm H_2_O ± 7.48; *p* = 0.005). Six (60%) repairs using the novel device had everted edges and three (30%) repairs using the conventional method had everted edges. While the difference was not statistically significant (*p* = 0.370) it is a component of a higher quality of repair. Lack of gaps also indicate a higher quality repair. Nine (90%) repairs using the novel device method had no gaps and four (40%) repairs using the conventional method had no gaps. The difference neared statistical significance (*p* = 0.057). There was notably less variation in the results of the repair pressure with novel device, indicating that the novel device was able to more reproducibly create a repair approaching water-tightness. Nerve root entrapment did not occur with the novel device, whereas 3 surgeons entrapped nerve roots with the conventional repair technique (Novel: 0% entrapment, Conv.: 30% entrapment; *p* = 0.016) demonstrating that the novel device is safer in this regard (Table 3).

**Table 3:** Novel dural repair device.

**Quality of repair metric**	**Novel dura device**	**Conventional suture technique**	***P*-value**
Water tightness	22.9 cm H_2_O ± 5.26	13.3 cm H_2_O ± 7.48	*p =* 0.005
Everted edges	60%	30%	*p =* 0.370
Entrapped nerve roots	0%	30%	*p =* 0.016

### Part C

The authors reported that the novel device was easy to use intraoperatively and that there was a perceptible time savings as illustrated by the benchtop evaluation. The ability to move the blunt tip of the device across the inside of the flap of the defect without creating damage and safely avoiding nerve roots, enabled surgeons to efficiently optimize the location of the suture passage. The authors found that these unique attributes accelerated the closure of accessible tears, and perhaps more importantly, offered the ability to safely and efficiently close tears that would otherwise be inaccessible with conventional methods.

In at least one instance, a challenging durotomy was encountered through a long tubular retractor that would have necessitated transitioning to an open exposure if the novel device had not been available; the surgeon felt that the repair would not have been possible with conventional instruments. The use of a single instrument to deploy suture through a tubular retractor eliminated the need for hand pronation or supination which would have been technically difficult or impossible in the confined space afforded by the retractor. Knot tying was facilitated by a suture pusher included with the novel device, which minimized the number of instruments required to handle suture within the tube, facilitating a technique familiar to surgeons with experience in arthroscopic knot tying. This example is one of multiple scenarios that was not possible to capture during the benchtop evaluation due to the standardization of the tear location and exposure, but which yielded a significant time savings and substantially reduced morbidity relative to alternative options intraoperatively.

The authors found that the benchtop results were qualitatively confirmed by the clinical experience. However, limitations in the benchtop portion of the evaluation failed to capture some of the most clinically relevant improvements experienced intraoperatively, such as repairing a tear with the novel device that would conventionally be inaccessible.

## Discussion

**Figure 2:**
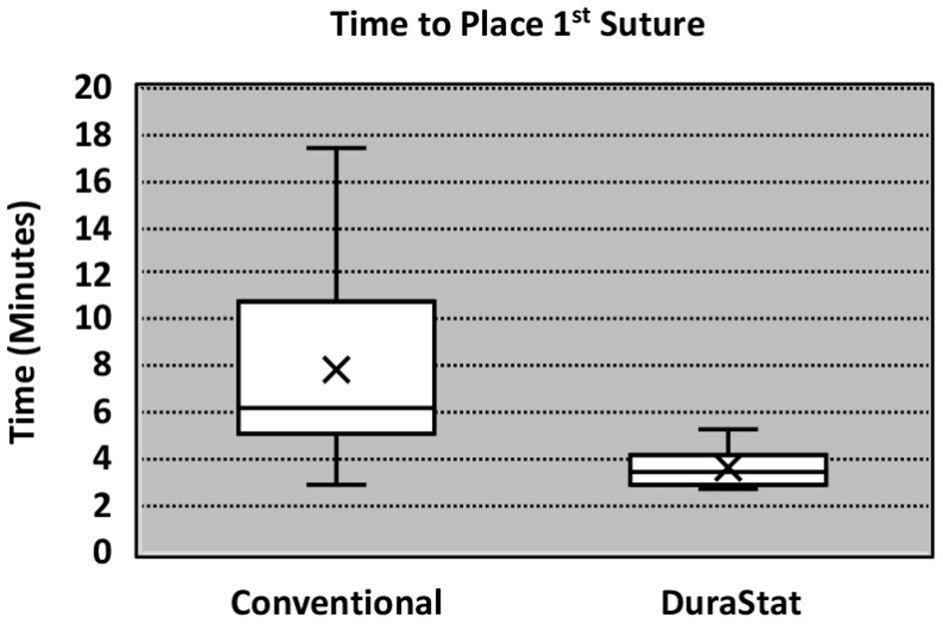
Boxplot of time to place 1st suture.

**Figure 3:**
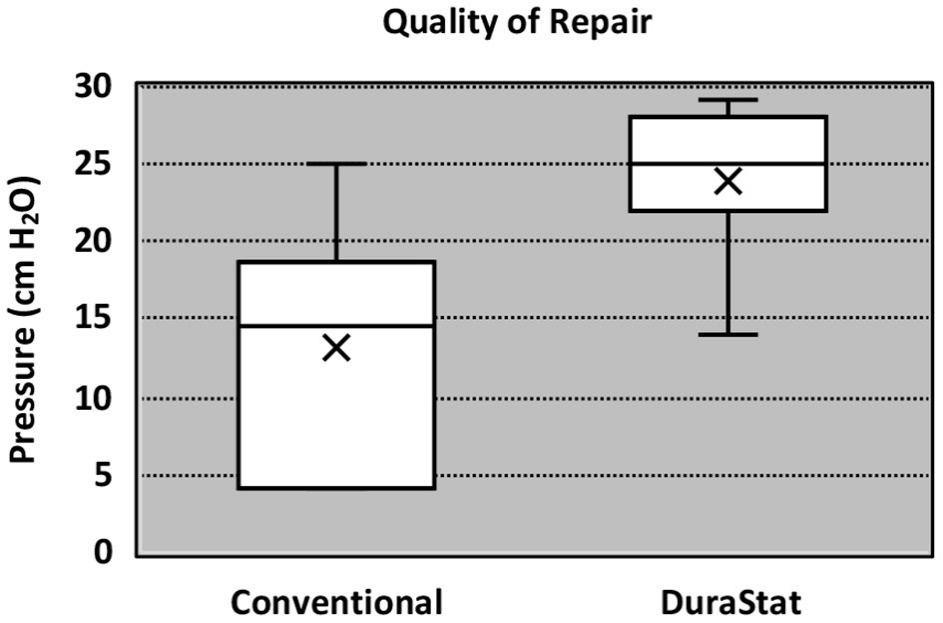
Boxplot of quality of repair.

Incidental durotomies are common during spine surgery and present a particular challenge in minimally invasive surgery which may necessitate conversion to an open technique ultimately leading to longer surgical times. A poorly repaired dural tear may lead to persistent CSF leak that may result in a return to the operating room. The goal of any dural repair technique is to provide an easy and efficient water-tight closure that will serve as a permanent solution. The novel dural repair device allows surgeons of all training levels to successfully repair a simulated dural tear faster, with higher quality, and without entrapping nerve roots, as compared to conventional techniques. The results of this study demonstrate that the novel device not only allows for a safe, fast and efficacious repair but it also enhances the reproducibility across surgeons, regardless of experience, dexterity, or skill level. The narrower interquartile range seen in Figures 2, 3 equates to consistent replication of the qualities of repairs that are essential in preventing persistent CSF leaks, re-operations, increased length of stay and the associated additive procedural expenses. Furthermore, these improvements were confirmed by the authors when this novel technology was employed in the operating room.

With respect to quality of repair, the inspection of the simulated dura samples revealed potential drivers of enhanced water-tightness. Aligned approximated tissue with everted edges, resulted in less bunching and gapping, suggesting that the increased repair quality and pressure is linked to the surgeon's ability to accurately pass each needle in a precise position which we theorize is due to the device's design and needle deployment control. Six (60%) repairs using the novel method had everted edges compared to three (30%) in the conventional method group. Another observed indicator which may be associated with a higher quality repair was the lack of gaps. Nine (90%) repairs using the novel method had no gaps and four (40%) repairs using the conventional method had no gaps. In practice, the novel device enables surgeons to more precisely select each position for suture passage, resulting in fewer suture needle holes and decreasing potential compromise to the dura.

As with any simulated surgical environment used for comparative testing, rigidly controlling variables has inherent limitations. Clinically, surgeons encounter factors not reflected in the model that introduces challenges leading to increased difficulty and time needed for repair. This includes external factors such as the availability and condition of dural repair instruments and the availability of suturing materials. Clinical factors such as inaccessibility of a laterally positioned tear, irregularly shaped tears, varied tissue quality, and intensified concerns as surgeons work around delicate structures are impossible to reproduce in a simulation. Additionally, the simulated dura itself is dissimilar in many qualities from real dura; e.g., human dura is much thinner. Specific to this study, the relatively small sample size may also be considered a limiting factor.

From the perspective of surgeon education and training, the simulated durotomy model serves as a viable teaching tool to reduce the learning curve associated with dural repair. The model emulates important elements such as depth, CSF flow, bleeding and nerve root herniation to mimic the surgical environment, and the direct pressure readings of the dural sac provides a standardized measure for repair quality. Despite the limitations of the model, there is value for surgeons to have access to the dural repair simulator on an ongoing basis.

Given the aforementioned inherent limitations of the simulated environment, the authors used the novel device clinically on numerous occasions. Perhaps the most obvious application for the novel device is through a minimally invasive surgery using a tubular retractor. In one such instance a surgeon encountered a 1.5 cm midline tear down a 9 cm long tubular retractor during a TLIF on an obese patient. The tear was unreachable using traditional tools and was too large to consider an adjunct without a primary repair. The novel dural repair device was better able to gain control of the tear and complete a primary suture repair. This was facilitated by the novel instrument's optimized length, bayoneting of the shaft to preserve visualization, and the deployment mechanism which releases the needle in an upward arc toward the surgeon's field of view. Both the needle and the attached suture are contained within the tip of the novel device which prevented the possibility of unintentional or premature perforation of the dura, or damage to surrounding tissues, as the surgeon found the optimal position for passage. This particular instance would have necessitated substantial additional operative time to expand the existing exposure to visualize (7) and repair the tear.

Durotomies encountered in the lateral recess and near the axilla pose another challenge for repair. Use of this novel technology makes these types of repairs, even in the open case setting, more feasible and potentially safer. When these situations were encountered intraoperatively on multiple occasions, a perceptible time savings and reduced difficulty was noted through the use of the novel device. In other clinical instances where the tear was more accessible, the novel device was used to create an initial closure of the dura efficiently, and with more precisely aligned edges. As the data indicated in Part A of the assessment, the first pass of the needle was much more efficient with the novel device to create the initial closure with the added benefit of properly aligning the edges to help achieve a water-tight closure, as illustrated in Part B. In some instances, the authors started the repair with the novel device to gain control of the tear, then augmented the repair with more conventional repair techniques. With this hybrid technique, the sutures from the first pass with the novel device may be used to manipulate the dura away from underlying nerve roots to facilitate safer and more efficient conventional suturing. The novel device has distinct advantages when tears occur in confined spaces or in difficult locations such as at the lateral recess where control of the dura and underlying nerve roots can be challenging. The novel device allows surgeons to minimize surgical time, find a safe tissue layer, and move the tip of the device into position without puncturing the tissue prematurely. Then, while in the ideal position, and after confirming nerve roots are clear, the needle can be predictably and reproducibly passed.

## Conclusion

When incidental durotomies occur in confined spaces the use of the novel dural repair device allows surgeons to complete difficult repairs in less time, with greater precision, higher quality and with enhanced patient safety. These attributes were quantifiable in two independent surgical simulations using an intricate wet model. When used clinically, the advantages of the novel repair device were confirmed both in minimally invasive and open procedures, resulting in enhanced patient outcomes. The novel device should be considered a viable tool to consistently reduce dural repair times, prevent persistent CSF leaks and obviate the need for additional tissue disruption to access to tears. The model created for the simulation has ongoing utility for surgeon training and skill development.

## Data Availability Statement

The original contributions presented in the study are included in the article/supplementary materials, further inquiries can be directed to the corresponding author/s.

## Author Contributions

CD was involved in simulation and clinical experiments as well as in editing manuscript. ES and KK was involved in original manuscript writing and editing. SP was involved in original manuscript writing, editing, and final revision. All authors contributed to the article and approved the submitted version.

## Conflict of Interest

This study was funded in part by DuraStat LLC for open-access publication fees. The materials for the benchtop portion of this investigation were also provided by DuraStat.
